# One-Step Synthesis of Nitrogen-Doped Porous Biochar Based on N-Doping Co-Activation Method and Its Application in Water Pollutants Control

**DOI:** 10.3390/ijms232314618

**Published:** 2022-11-23

**Authors:** Yingjie Su, Yuqing Shi, Meiyi Jiang, Siji Chen

**Affiliations:** 1 College of Life Sciences, Jilin Agricultural University, Changchun 130118, China; 2 Key Laboratory of Straw Comprehensive Utilization and Black Soil Conservation, Ministry of Education, Jilin Agricultural University, Changchun 130118, China

**Keywords:** one-step synthesis, N-doping co-activation, biochar, water pollutants, adsorption performance

## Abstract

In this work, birch bark (BB) was used for the first time to prepare porous biochars via different one-step methods including direct activation (BBB) and N-doping co-activation (N-BBB). The specific surface area and total pore volume of BBB and N-BBB were 2502.3 and 2292.7 m^2^/g, and 1.1389 and 1.0356 cm^3^/g, respectively. When removing synthetic methyl orange (MO) dye and heavy metal Cr^6+^, both BBB and N-BBB showed excellent treatment ability. The maximum adsorption capacities of BBB and N-BBB were 836.9 and 858.3 mg/g for MO, and 141.1 and 169.1 mg/g for Cr^6+^, respectively, which were higher than most previously reported biochar adsorbents. The probable adsorption mechanisms, including pore filling, π–π interaction, H-bond interaction, and electrostatic attraction, supported the biochars’ demonstrated high performance. In addition, after five recycles, the removal rates remained above 80%, which showed the high stability of the biochars. This work verified the feasibility of the one-step N-doping co-activation method to prepare high-performance biochars, and two kinds of biochars with excellent performance (BBB and N-BBB) were prepared. More importantly, this method provides new directions and ideas for the development and utilization of other biomasses.

## 1. Introduction

Water pollution is a serious threat to the ecosystem and human safety [1]. Organic pollutants (aromatic species) and heavy metals in water have become the focus of global attention due to their strong teratogenicity, carcinogenicity, and non-degradability [2,3]. Cr^6+^ is one of the main forms of Cr. Because of its high solubility, easy accumulation, and difficulty degrading in the ecosystem, Cr^6+^ is one of the main toxic heavy metals that pollute water bodies [4]. Cr^6+^ ions are discharged into the water in large quantities during industrial processes such as metallurgy and electroplating [5]. Similarly, synthetic dyes are widely used in the printing, dyeing, and textile industries [6]. Many synthetic dyes have caused irreversible threats to human safety, such as skin allergy and cancer, and have become a research hotspot in the field of water pollution remediation [7,8]. Therefore, it is important to remove Cr^6+^ and synthetic dyes to purify water.

At present, the removal methods for Cr^6+^ and synthetic dyes from polluted water mainly include biological treatment, chemical treatment, and physical treatment [9,10,11]. As one of the physical treatment methods, the adsorption method is widely used in water pollution treatment because of its low cost, lack of by-products, and mild conditions [12,13]. It is also considered an ideal method to effectively remove Cr^6+^ and synthetic dyes. Many materials, such as graphene oxide and carbon nanotubes, are used to treat water pollution due to their excellent adsorption properties on pollutants, but the high cost often limits their application [14,15]. In contrast, biochar is attracting increasing attention as an excellent adsorbent with a large specific surface area, abundant functional groups, and a well-developed pore structure [16,17]. Unfortunately, due to the limited adsorption sites, the adsorption performance is often limited. Appropriately modifying and improving the adsorption efficiency of biochar is the key to determining whether it can become a widely used adsorbent.

As an effective strategy to improve biochar performance, nitrogen doping (N-doping) technology has been applied in environmental remediation, such as adsorption, catalysis, and electrochemistry [18,19]. N hetero-atoms provide more binding sites on the biochar surface, which enhances the hydrophilicity and adsorption capacity of the compound by forming a more stable complex with pollutants [20]. At the same time, the N-doping method can adjust part of the electronic structure in the carbon skeleton, and further improve the reactivity of the biochar [21]. The pore structure, specific surface area, and other physical and chemical properties of biochars are also crucial factors that not only affect the adsorption capacity but also determine the degree of adsorbed pollutant diffusion [22,23]. The pore-forming effect of common activators (such as NaOH and KOH) in biochar preparation is beyond doubt [24,25]. However, there are few studies on biochar prepared with one-step pyrolysis and N-doping.

To remedy this, we explored a novel biochar preparation strategy to improve the performance of biochars, that is, N-doping co-activation. N-doping co-activation refers to the incorporation of nitrogen sources in the activation process in order to complete nitrogen doping and activation at the same time and successfully prepare nitrogen-doped porous biochars in one step. In this study, Birch bark was used as the carbon source for biochar preparation for the first time. N-doped porous biochar was synthesized with one-step co-activation pyrolysis by adding an activator (NaOH) and a nitrogen source (urea) in the preparation process. The focus of this study is to determine the feasibility of one-step N-doping co-activation in biochar preparation and its superiority in water pollution treatment, in order to provide a more effective way to deal with water pollution.

## 2. Results and Discussion

### 2.1. Preparation of CBB, BBB, and N-BBB

**Figure 1:**
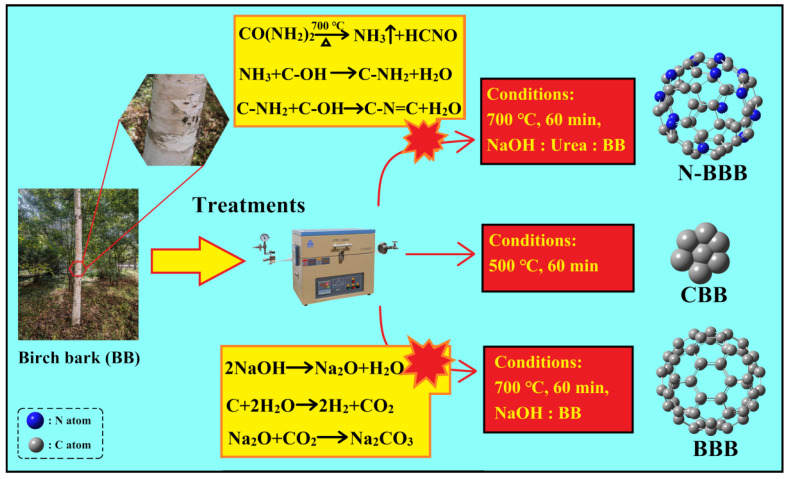
Schematic diagram of the preparation.

The sample preparation process is shown in Figure 1 and can be divided into three processes: direct carbonization, activation, and N-doping co-activation. The probable chemical reactions during the processes are summarized as follows:

*Activation process* [26,27,28]: In the high-temperature pyrolysis process, NaOH will generate Na_2_O and water, and then be further ionized to form Na^+^ and OH^−^. These ions will migrate or insert carbon precursors and react with them to generate carbon dioxide and water. The carbon dioxide further reacts with the oxide produced by NaOH (Na_2_O) to form carbonate (Na_2_CO_3_), which etches the carbonized sample. Finally, microporous, or mesoporous, structures are formed in the carbon.

*N-doping process* [29,30,31]: The decomposition of CO(NH_2_)_2_ at a high temperature produces solid and gaseous substances. Through the diffusion of the gas, the surface of the biochar develops various wrinkles and pores. The NH_3_ released during the pyrolysis process combines with the -OH group in lignocellulose (the main component of BB) to form C-NH2, and further generate C=N-C [20]. Eventually, these nitrogenous groups form various N-bond configurations including pyrrolic-N, pyridinic-N, and graphite-N.

### 2.2. Results of Characterizations

**Figure 2:**
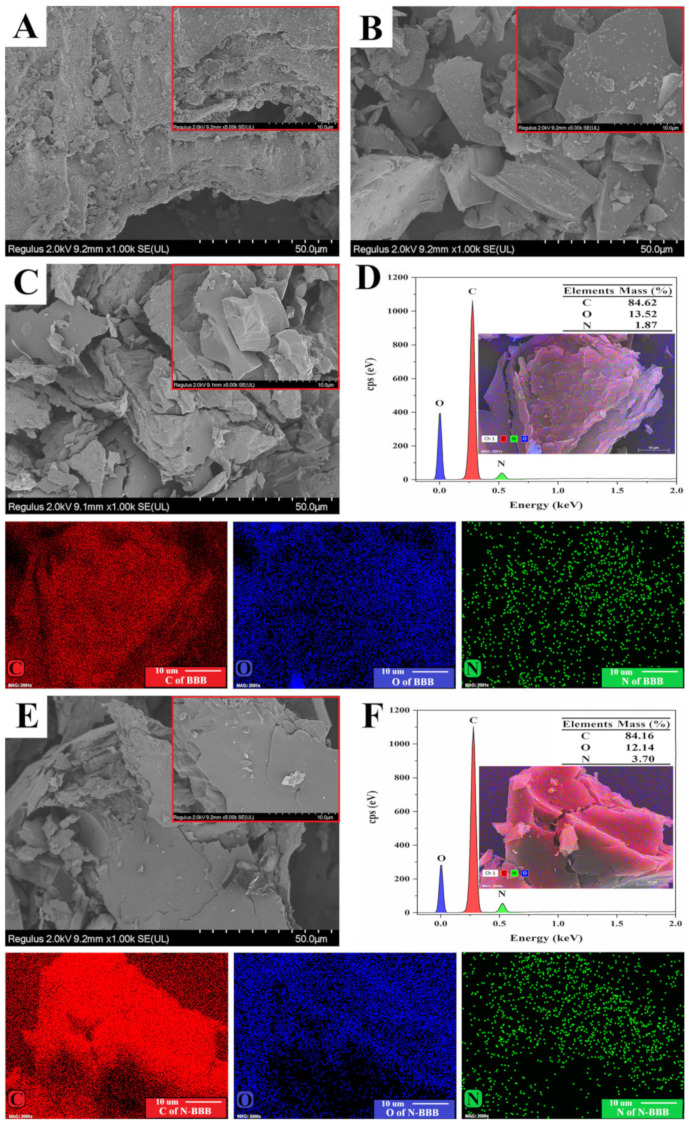
SEM images of (**A**) BB, (**B**) CBB, (**C**) BBB, and (**E**) N-BBB. EDS mapping of (**D**) BBB and (**F**) N-BBB.

The microscopic morphology and elemental composition of the samples were observed by SEM and EDS, as shown in Figure 2. BB had a rough, irregular surface and uneven lamellar structure. After carbonization (Figure 2B), CBB began to show a dense and smooth surface morphology. This can be explained by the fact that the main composition of BB is lignocellulose, consisting mainly of cellulose composed of glucose and hemicellulose composed of xylose [32]. During the high-temperature pyrolysis process, denatured glucose and xylose changed the morphology of CBB [33]. With the activation process treatment, especially in the presence of NaOH, BBB, and N-BBB (Figure 2C,E), many cracks and fragmentation occurred on the surface due to a severe reaction phenomenon. BBB and N-BBB were mainly composed of C, O, and N elements. However, because of different treatments, their nitrogen content was different. The C, O, and N contents of BBB and N-BBB were 84.62%, 13.52%, and 1.87%, and 84.16%, 12.14%, and 3.70%, respectively. Compared with BBB, the content of N in N-BBB nearly doubled. The above results not only proved the success of the activation process but also proved the feasibility of the N-doping co-activation process.

**Figure 3:**
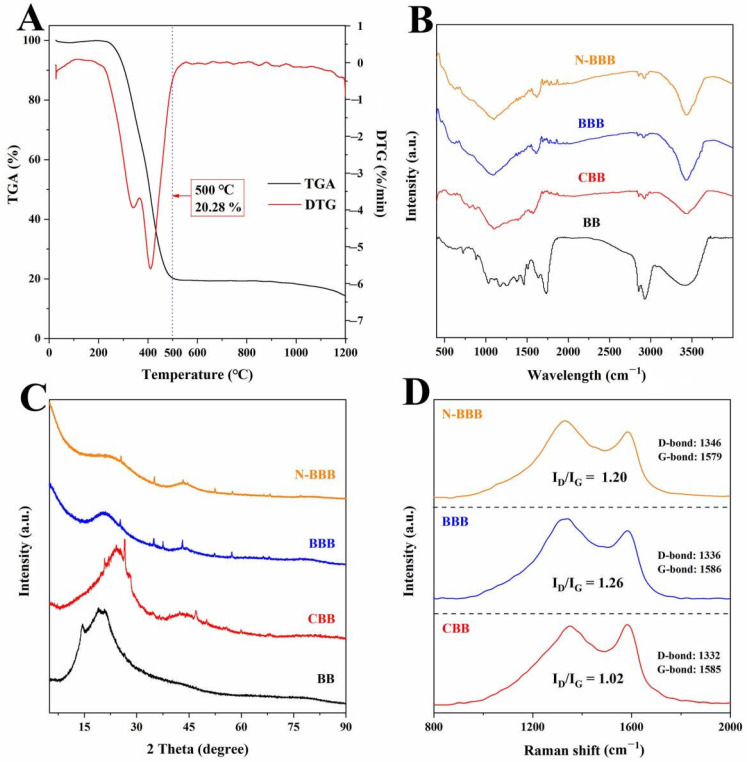
(**A**) TGA curves of BB. (**B**) FT-IR spectra and (**C**) XRD of BB, CBB, BBB, and N-BBB. (**D**) Raman spectra of CBB, BBB, and N-BBB.

The influence of temperature on BB was measured by a TGA test under N_2_ protection, as shown in Figure 3A. The TGA curves corresponding to three main stages of weight loss ranged from room temperature to 1200 °C. The first stage occurred at room temperature to 275 °C and was caused by evaporation and loss of residual water from physical surfaces and internal pores [34,35]. The second stage, which started at 275 °C and ended at 500 °C, was the most significant stage of weight loss. It can be explained by the fact that the main oxygenated component of BB was lignocellulose, which can be cracked into gases and tar at higher temperatures. With the removal of these pyrolytic substances, weight loss in the second stage was induced. Therefore, 500 °C was selected as the carbonization temperature for CBB preparation, and the yield was 20.28% at this time. As for the third stage (500–1200 °C), the TG curve was relatively stable, indicating that there was no obvious weight loss phenomenon. However, when the temperature exceeded 1000 °C (especially when it was close to 1200 °C), CBB exhibited a slight mass change, which may have been caused by the pyrolysis of some minerals [36,37].

The FT-IR spectra of the functional groups of the samples were analyzed, as shown in Figure 2B. The broad band at around 3450 and 2930 cm^−1^ represented the stretching vibrations of hydroxyl functional groups (O-H) and -CH, -CH^2^, and -CH^3^ groups [38]. The band around 1730 cm^−1^ represented the stretching vibration of C=O. The peak at 1630 cm^−1^ represented the axial deformation of the carbonyl group. The band at around 1375 and 1420 cm^−1^ represented the C-H symmetric bending vibration of the methyl group and the deformation vibration of methylene [38,39]. In common with many biomass materials [34,35,38,39], the bands at around 1047–1159 cm^−1^ represented the tensile vibrations of C-O from alcohols, phenols, acids, or esters. After N-doping co-activation, a new vibration peak appeared at 1690 cm^−1^, which indicated that the C=N was formed on N-BBB [20].

The crystal structure of the samples was tested by XRD, as shown in Figure 3C. The peaks at 17° and 23° represent the cellulose from the lignocellulose of raw BB [32,33]. The irregular peaks represent minerals that can be interpreted as inorganic salts. These peaks became sharper and more distinct after carbonization. After direct activation and N-doped co-activation, these peaks were significantly weakened, which can be explained by the removal of a large quantity of soluble mineral salts during the washing process. In addition, we noted that the diffraction peaks of CBB, BBB, and N-BBB were in the range of 10–30° and 38–45°, indicating that the prepared biochars had the local structure of typical carbon materials; that is, they contained both amorphous carbon and a 2D graphite planar structure [40].

The presence of defects in the carbon was determined by Raman spectra, as shown in Figure 3D. Two typical peaks obtained from the results include the D-band with amorphous carbon at around 1339 ± 7 cm^−1^ and the G-band with graphitic carbon at around 1583 ± 3 cm^−1^ [38,39,40]. To measure the degree of defect and disorder in the carbons, the intensity ratio of the D-band and G-band (I_D_/I_G_) was used as an important index. The I_D_/I_G_ value of CBB was 1.02. After activation, the I_D_/I_G_ value of BBB and N-BBB were 1.26 and 1.20, which indicated that more amorphous carbon structures were generated in the biochars.

**Figure 4:**
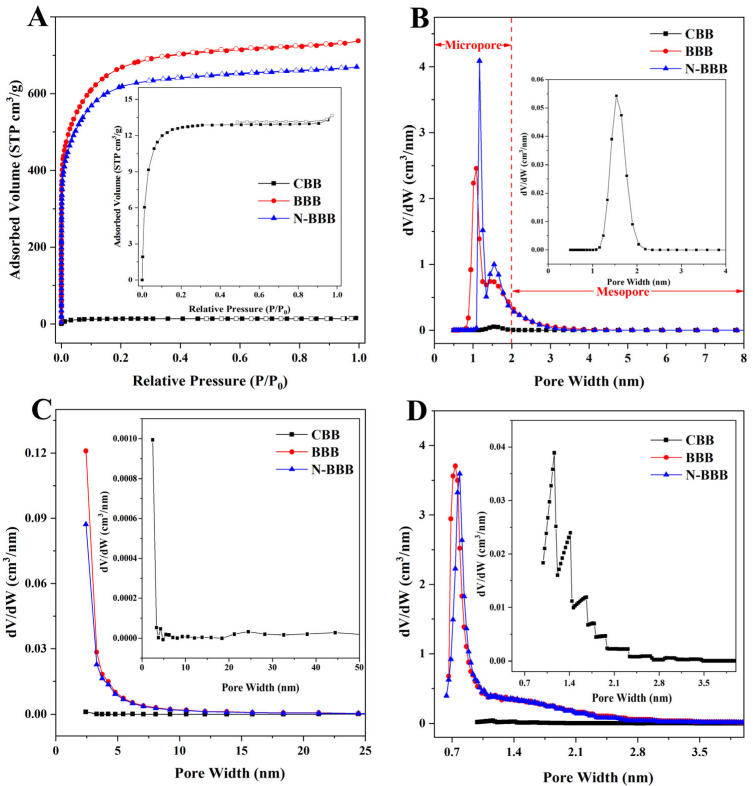
(**A**) N_2_ adsorption-desorption isotherms of samples. Pore distribution of samples based on (**B**) NLDFT method, (**C**) BJH method, and (**D**) H-K method.

The specific surface area and porosity of the samples were tested by N_2_ adsorption-desorption isotherms, as shown in Figure 4, and the data are shown in Table 1. The specific surface area and the total pore volume of CBB were 49.5 m^2^/g and 0.0222 cm^3^/g, respectively, which were not enough to support CBB as a porous biochar for adsorption. Therefore, further activation treatment is needed to greatly improve its properties and enhance its application performance. After direct activation and N-doping co-activation, the specific surface areas of BBB and N-BBB were 2502.3 and 2292.7 m^2^/g, respectively, and their total pore volumes were 1.1389 and 1.0356 cm^3^/g, respectively. Compared with CBB, the values were greatly improved, which indicated the success and effectiveness of the further activation treatment. Moreover, both BBB and N-BBB showed typical type IV isotherms with a slight H3 hysteresis loop, indicating that some mesoporous structures existed in the prepared biochars [39,40]. The volumes of micropores in BBB and N-BBB were 1.1118 and 1.0170 cm^3^/g, respectively, accounting for 97.6% and 98.2% of the total pore volume, respectively, indicating that they were porous biochars with mainly micropore structure and partly mesoporous structure [24,25]. Pore size distribution was used to further analyze the porosity of the samples. The results based on the NLDFT method also showed that both BBB and N-BBB had microporous and mesoporous structures. The BJH and H-K methods were used to study the pore distribution, which not only re-analyzed the multi-pore structure of BBB and N-BBB but also further proved the existence of both mesoporous and microporous structures.

In addition, it is noteworthy that the specific surface area and total pore volume of N-BBB were smaller than those of BBB. There are three possible reasons for this. First, the addition of the nitrogen source urea in the activation process will compete with the activator NaOH for the contact area with the carbon precursor, thus affecting the specific surface area and pore structure of N-BBB. Second, it may be caused by the chemical reaction between the ammonia gas generated by the nitrogen source urea at high temperature and carbon dioxide produced in the activation process and water vapor, thus consuming part of the activator and reducing the activation efficiency. Third, the nitrogen source urea and activator simultaneously etched the surface of the carbon material and generated more gases [29,30,31], which diffused into the carbon precursor, resulting in partial fragmentation, affecting the pore structure and resulting in the reduction in the specific surface area and total pore volume.

**Figure 5:**
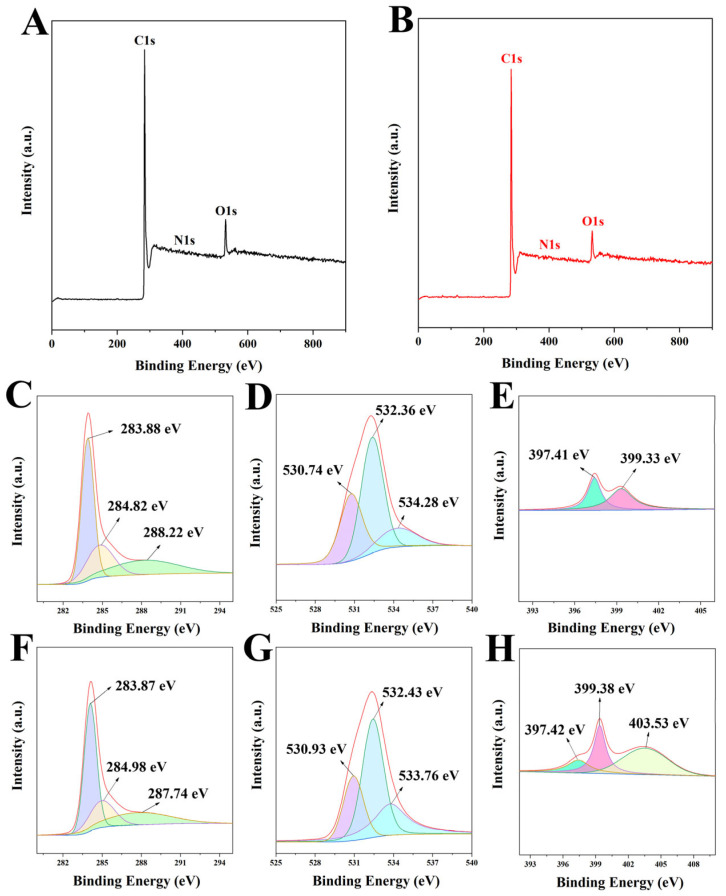
XPS spectra of (**A**) BBB and (**B**) N-BBB. The C1s, O1s, and N1s high-resolution spectra of BBB (**C**–**E**) and N-BBB (**F**–**H**).

The surface chemical and electronic states of the samples were determined by XPS spectra, as shown in Figure 5. Both BBB and N-BBB contained mainly C, O, and N elements, which was consistent with the EDS. The high-resolution C1s spectra of BBB and N-BBB showed three classical peaks at 283.87–283.88, 284.82–284.98, and 287.74–288.22 eV corresponding to C-C, C-O, and C=O, respectively [38,39,40]. The high-resolution O1s spectra of BBB and N-BBB both had three peaks at 530.74–530.93, 532.36–532.43, and 533.76–534.28 eV corresponding to C=O, C-O, and -OH, respectively [38,39,40]. The high-resolution N1s spectra of ITGB and MITGB both had peaks at 397.41–37.42 and 399.33–399.38 eV, corresponding to pyridinic-N and pyrrolic-N, respectively [38,39,40]. In addition, N-BBB had a unique peak corresponding to graphite-N at 403.53 eV [20], indicating that the N-doped co-activation method had indeed successfully doped N into N-BBB.

### 2.3. Results of Adsorption Performances

#### 2.3.1. Adsorption Kinetics

**Figure 6:**
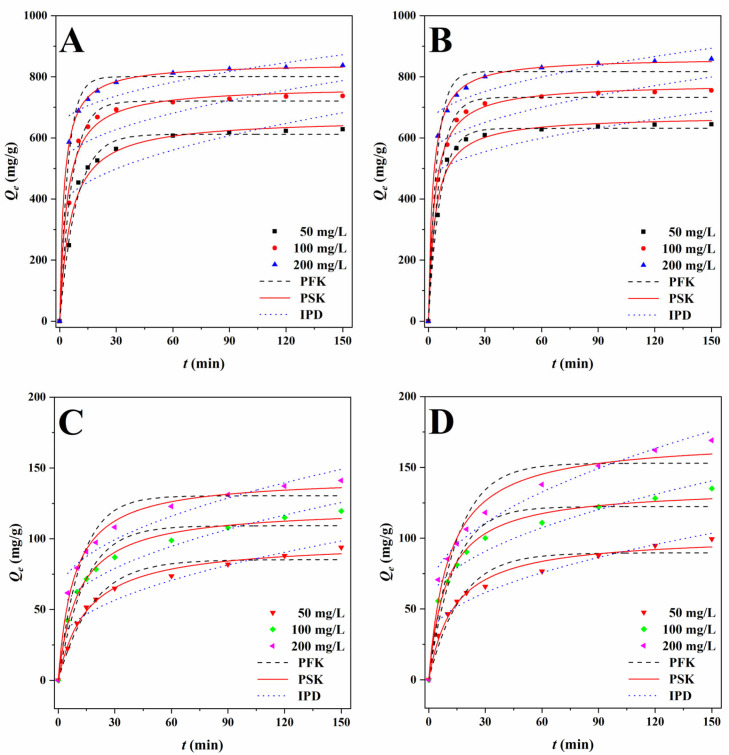
PFK, PSK, and IPD plots of MO for (**A**) BBB and (**B**) N-BBB, and plots of Cr^6+^ for (**C**) BBB and (**D**) N-BBB at 303 K.

Adsorption kinetics describes the adsorption capacity of the adsorbent at different initial solution concentrations as a function of contact time [32,33,34,35]. Therefore, the effect of time on the adsorption of MO and Cr^6+^ by BBB and N-BBB at a temperature of 303 K was explored, as shown in Figure 6. Whether the adsorbent was BBB or N-BBB, or the adsorbate was MO or Cr^6+^, all the adsorption process trends were similar. The adsorption capacities increased sharply in the first 30 min, and then gradually reached equilibrium at 60 min. The extension of contact time did not further significantly improve the adsorption capacities. It can be speculated that the adsorption capacities would increase with the increase in the initial concentration of the solution, and the high concentration of solution promoted the adsorption process to some certain extent [40]. In order to study the control mechanism of reactions in the process of adsorption, three common adsorption kinetic models, Lagergren’s PFK model based on surface physical adsorption [40], Ho–McKay’s PSK model based on chemical adsorption [39], and Weber–Morris’s IPD model based on molecular diffusion [38] were used to analyze the experimental data, as shown in Table 2.

The PFK correlation coefficients *R*^2^ of BBB for MO ranged from 0.9780 to 0.9944; while the *Q_e.cat_* values (611.8, 720.4, and 800.3 mg/g) were lower than the *Q_e_* (627.9, 737.2, and 836.9 mg/g) obtained from the experiments. The PFK for Cr^6+^ ranged from 0.9550 to 0.9741; while the *Q_e.cat_* values were 85.3, 109.2, and 130.4 mg/g for different initial concentrations, and were again lower than *Q_e_* (93.8, 119.6, and 141.1 mg/g). To summarize, the PFK model was not the best kinetic to describe the whole adsorption process. The IPD correlation coefficients *R*^2^ of BBB for MO ranged from 0.5822 to 0.7517, indicating that the adsorption process of MO by BBB may not be affected by particle diffusion. For Cr^6+^, on the contrary, the correlation coefficients *R*^2^ ranged from 0.9138 to 0.9280, which indicated the adsorption process had a particle diffusion behavior. Ho–McKay’s PSK model was used to fit the data; the PSK correlation coefficients *R*^2^ of BBB ranged from 0.9848 to 0.9997 for MO and from 0.9913 to 0.9919 for Cr^6+^. Meanwhile, the *Q_e.cat_* values (664.5, 769.0, and 844.2 mg/g for MO, and 98.4, 122.3, and 143.6 mg/g for Cr^6+^) agreed with those obtained from experiments, which showed the applicability of PSK in the adsorption process. 

When the models were used to fit with the data of N-BBB, the PFK correlation coefficients *R*^2^ were 0.9721–0.9952 for MO and 0.9318–0.9506 for Cr^6+^. The *Q_e.cat_* values were 631.3, 732.9, and 817.1 mg/g for MO, and 89.7, 122.4 and 153.0 mg/g for Cr^6+^, which were both lower than the *Q_e_* values (644.4, 755.1 and 858.3 mg/g for MO, and 99.5, 135.0, and 169.1 mg/g for Cr^6+^) obtained from experiments. It can be speculated that the PFK model may have played a role in the adsorption process, although the role was not dominant. For the IPD model, the correlation coefficients *R*^2^ were 0.5471–0.7830 for MO and 0.9432–0.9746 for Cr^6+^, also indicating that the adsorption process of N-BBB for Cr^6+^ had a particle diffusion behavior and was largely unaffected by particle diffusion for MO. The PSK coefficients *R*^2^ were 0.9873–0.9989 for MO and 0.9765–0.9869 for Cr^6+^, indicating the PSK model was more suitable to describe the adsorption process. Moreover, it was found that, with the increase in the initial concentration of the solution, the rate constants *k*_2_ of BBB were also increased for both MO and Cr^6+^, indicating that the adsorption rate gradually accelerated, and the adsorption rate was faster at a higher concentration. However, the rate constants *k*_2_ of N-BBB were different. For MO, N-BBB showed a similar trend to BBB. At the same time, the rate constants *k*_2_ of N-BBB for Cr^6+^ decreased gradually, which indicated that, with the increase in initial concentration, the adsorption rate gradually slowed, and the adsorption rate was slower at a higher concentration.

According to the results, we inferred that the adsorption processes of BBB and N-BBB for MO and Cr^6+^ may be mainly chemical reactions (while physical adsorption and particle diffusion also had certain effects on the adsorption process), and the adsorption behaviors between adsorbent and adsorbate through transfer, exchange, or sharing to form chemisorption bonds, may control the adsorption rate [40,41].

#### 2.3.2. Adsorption Isotherms

**Figure 7:**
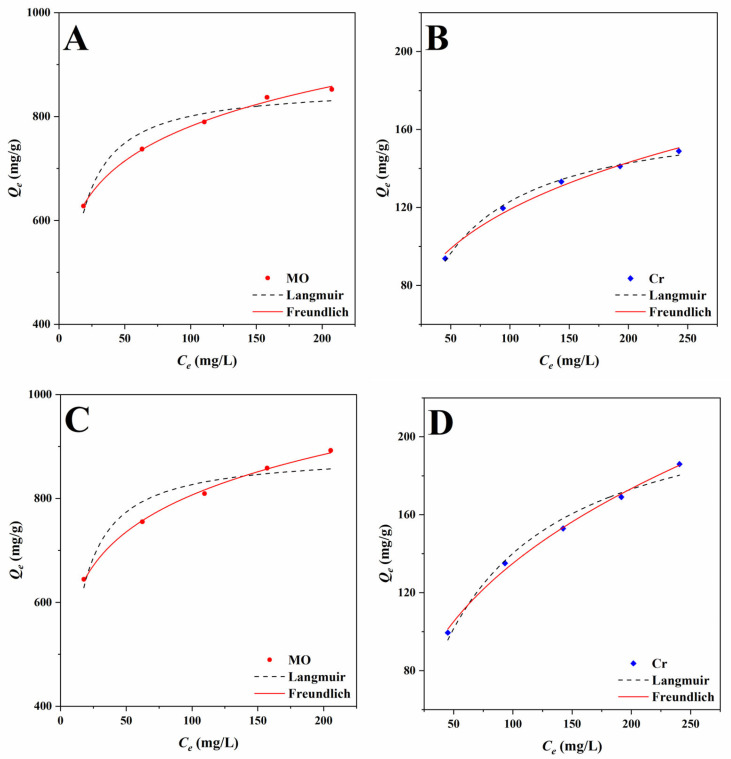
Adsorption isotherms of MO and Cr^6+^ for BBB (**A**,**B**) and N-BBB (**C**,**D**) at 303 K.

The effect of concentration on the adsorption capacity of the adsorbent is usually determined by investigating the adsorption isotherms. The effects of different initial concentrations of solution on the adsorption process of BBB and N-BBB were studied at a temperature of 303 K, and the results are shown in Figure 7. Increasing the initial concentration of MO or Cr^6+^ solution was beneficial to the forward process of adsorption. 

Subsequently, the Langmuir and Freundlich isotherm models were used to analyze the experimental data, and the results are shown in Table 3. The Langmuir isotherm model is often used to describe the adsorption process of homogeneous molecules [42], while the Freundlich isotherm model is often used to study the heterogeneous multilayer adsorption process [43]. When the adsorbate was MO, the Langmuir isotherm correlation coefficients *R*^2^ were 0.9327 for BBB and 0.9041 for N-BBB, which indicated that the adsorption processes were not uniform single-layer. The *Q_m_* of BBB and N-BBB for MO were 860.3 and 887.4 mg/g, higher than *Q_e_*, indicating that the prepared biochars had higher adsorption capacities for MO. Moreover, the *K_L_* of N-BBB for MO was slightly bigger than that of BBB, which showed that N-BBB had a faster MO adsorption rate. The Freundlich isotherm correlation coefficients *R*^2^ for MO were 0.9967 for BBB and 0.9883 for N-BBB. The *n_F_* values of BBB and N-BBB for MO were 7.75 and 7.51, bigger than 1.0, indicating that this adsorption model of fitting MO was appropriate. As for Cr^6+^, the Langmuir isotherm correlation coefficients *R*^2^ were 0.9949 for BBB and 0.9822 for N-BBB, which also showed that the uniform single-layer adsorption was not suitable to describe the adsorption process. When the Freundlich isotherm was used to fit the data, the correlation coefficients *R*^2^ were 0.9976 for BBB and 0.9959 for N-BBB; while the *n_F_* values of BBB and N-BBB were 3.76 and 2.77. The results indicated that the adsorption processes were non-uniform multilayer [42,43,44].

#### 2.3.3. Adsorption Thermodynamics

**Figure 8:**
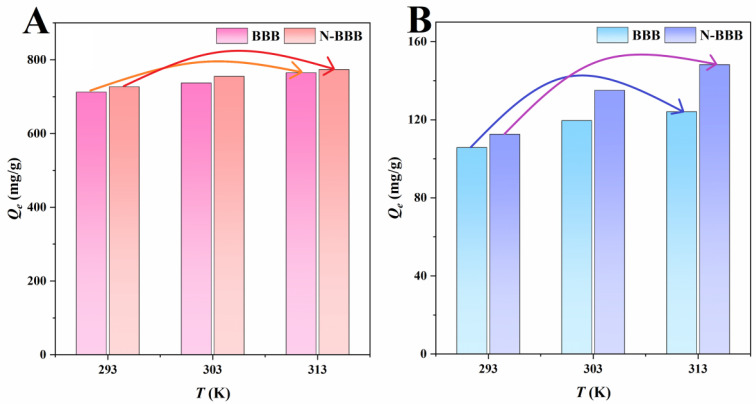
Adsorption thermodynamics of BBB and N-BBB for (**A**) MO and (**B**) Cr^6+^.

The influence of temperature (293, 303, and 313 K) on adsorption by BBB and N-BBB is shown in Figure 8. On increasing the temperature from 293 K to 313 K, the adsorption capacities of BBB for MO and Cr^6+^ increased from 712.5 to 764.8 mg/g and from 105.8 to 124.1 mg/g, respectively, while the adsorption capacities of N-BBB for MO and Cr^6+^ increased from 726.7 to 773.7 mg/g and from 112.6 to 148.3 mg/g, respectively. Obviously, raising the temperature increased the adsorption capacities of MO and Cr^6+^—that is, a high-temperature environment promoted the adsorption processes by biochars (BBB and N-BBB). 

The experimental data were analyzed by thermodynamics formulas, and the parameters are shown in Table 4. All Δ*G* values were negative, indicating that the adsorption occurred spontaneously, both with BBB (−5.86, −6.19, and −6.55 for MO and −0.27, −0.61, and −0.73 for Cr^6+^) and N-BBB (−5.93, −6.29, and −6.60 for MO and −0.43, −0.93, and −1.23 for Cr^6+^) [39,40]. The thermodynamic enthalpy values of Δ*H* of adsorption of MO were 4.29 and 3.82 kJ/mol for BBB and N-BBB, respectively, and the Δ*H* values of adsorption of Cr^6+^ were 6.47 and 11.23 kJ/mol for BBB and N-BBB, respectively, which further confirmed the endothermic property of the adsorption process [38]. In addition, the positive values of thermodynamic Δ*S* (34.61 and 33.27 Jmol^−1^ K^−1^ for MO, 23.01 and 39.78 J mol^−1^ K^−1^ for Cr^6+^) indicated that the randomness and chaos of the interface between the porous biochars and solutions increased with the increase in temperature [40].

#### 2.3.4. Effect of pH

**Figure 9:**
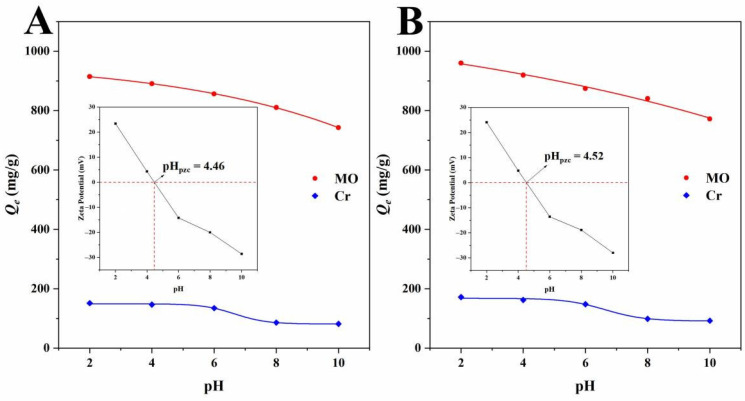
Effect of pH on the adsorption capacities of MO and Cr^6+^ onto (**A**) BBB and (**B**) N-BBB (inset: Zeta potential).

In general, pH affects the adsorption process by changing the charge properties of the adsorbent and the adsorbate [32,33,34,35]. MO has two chemical structures, basic and acidic, and whether the chromophore was anthraquinone or azo bond depends on the pH of the solution [45]. The adsorption of MO by biochars was investigated in the pH range of 2 to 10, and the results are shown in Figure 9A. With the increase in pH, the adsorption capacity of both BBB and N-BBB decreased, which can be explained by the electrostatic attraction between the surface charge of the biochars and the ionic charge of the anionic dye MO. Compared with BBB, N-BBB had higher electronegativity due to the N-doping. When the pH value was less than 6, the affinity of the dye increased, and the negative charge on the surface of the biochar could be used as the active site to generate a strong electrostatic attraction with the dye in solution. Conversely, when the pH was higher than 6 (particularly 8), the protonation of the dye was gradually weakened, and the biochars with negative active sites on the surface were not conducive to the adsorption of anionic dyes due to electrostatic repulsion, which reduced the adsorption amount of MO. Cr^6+^ also possessed various forms at different pH levels [4,5,20], where it exists as HCrO_4_^−^ and Cr_2_O_7_^2−^ when the pH is lower than 6.5, and as Cr_2_O_7_^2−^ and CrO_4_^2−^ when the pH is higher than 6.5. With the increase in pH, the adsorption capacities of both BBB and N-BBB decreased, and the maximum adsorption capacities were at a pH value of 2. This can be explained by the fact that the biochars (BBB and N-BBB) had a positive surface charge at a pH less than pH_pzc_ (4.46 for BBB and 4.52 for N-BBB). When the pH was higher than 6, the adsorption capacities decreased sharply, which may be due to electrostatic repulsion. When the pH was in the range of 8 to 10, the adsorption became relatively stable, which indicated that electrostatic interaction was not the only process affecting adsorption performance [20].

### 2.4. Results of Cycle Tests

**Figure 10:**
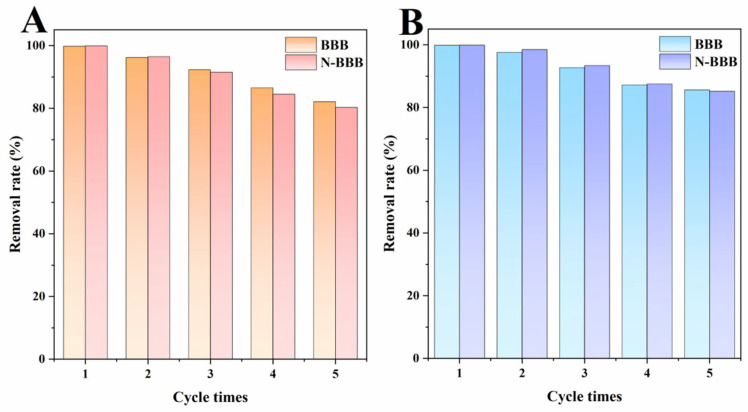
Cycling stability tests of BBB and N-BBB for (**A**) MO and (**B**) Cr^6+^.

The recyclability of adsorbents is an important parameter for evaluating the practical performance of biochars [38,39,40]; thus, the five-cycle performance of the biochars was investigated, and the results are shown in Figure 10. The removal rates of MO and Cr^6+^ by BBB and N-BBB decreased with an increase in cycle number. This can be explained as follows: on the one hand, with the treatment of the cycling experiment, adsorbed organic pollutants formed by-products on the surface of biochars [38,39]; while on the other hand, with the increase in the carbonization regeneration process, the structure of the biochars became more fragile, which further affected its regeneration. At the same time, after five cycles, the removal rate of MO and Cr^6+^ by BBB and N-BBB remained above 80%, indicating that they had good stability and regeneration ability.

### 2.5. Probable Mechanism Analysis

**Figure 11:**
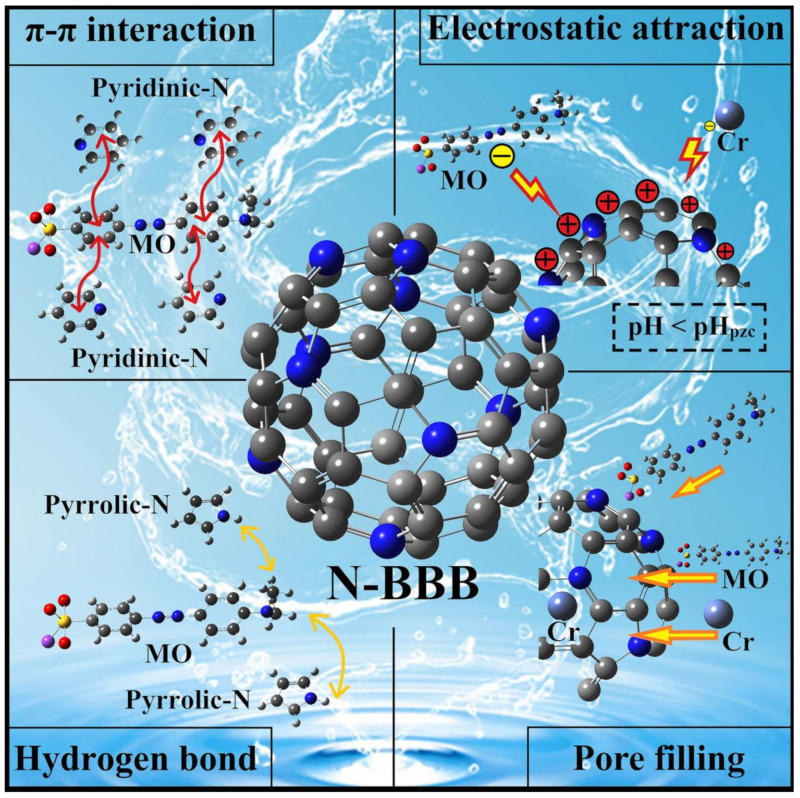
Probable mechanisms analysis for N-BBB removal of MO and Cr^6+^.

In this work, the N-BBB exhibited good removal ability of MO and Cr^6+^ in water solutions affected by many factors (Figure 11). Firstly, the large specific surface area and high total pore volume (2292.7 m^2^/g and 1.0356 cm^3^/g) of N-BBB provided many adsorption sites for the adsorption pollutant; therefore, it can be speculated that pore filling may play an important role in the adsorption process. The results based on the kinetics and isotherm showed that the adsorption process was heterogeneous multilayer adsorption with a chemical reaction, which indicated that the chemical binding force will also promote the adsorption process. In addition, from the test results with FT-IR and XPS, it can be speculated that many unsaturated functional groups containing carbon and oxygen on the biochar surface will produce hydrogen-bond interaction with the pollutant model. Moreover, FT-IR and Raman test results also showed that the prepared biochars contained aromatic rings and sp^2^ hybridized carbon with graphite structure, and the π bond in these structures may also have π–π interaction with aromatic rings in pollutants to enhance the adsorption capacity. The charged pollutants in the appropriate pH environment formed a strong electrostatic attraction with N-BBB, which further promoted the adsorption process. Experiments under different conditions, such as the initial concentration of the solution and the temperature of the adsorption process, show that these also affect the adsorption process. To summarize, in addition to the experimental conditions, the pore filling, π–π interaction, H-bond interaction, and electrostatic attraction supported the excellent performance of N-BBB.

### 2.6. Comparison

Adsorption capacity is an important parameter for evaluating the practical performance of an adsorbent; thus, the adsorption capacities of BBB and N-BBB are compared with other biochars, as shown in Table 5. The adsorption capacities of CBB for MO and Cr^6+^ were only 25.2 and 10.4 mg/g. After activation and N-doping co-activation, the adsorption capacities of BBB and N-BBB to MO and Cr^6+^ were significantly enhanced. In addition, compared with other biochars, the adsorption capacities of BBB and N-BBB were not low, which fully indicated that the prepared biochars had great potential and application prospects in the treatment of water pollutants.

## 3. Materials and Methods

### 3.1. Materials and Reagents

Birch bark (BB), *Betula Mandshurica Nakai*, obtained from the campus of Jilin Agricultural University (Changchun, China) in 2022, was washed with deionized water, dried at 80 °C for 12 h, and crushed. Urea, NaOH, H_2_SO_4_, HCl, and ethanol were purchased from Beijing Chemical Works (Beijing, China) and used without further purification.

Diphenylcarbazide (CAS: 140-22-7), Methyl orange (MO, CAS: 547-58-0), and Potassium dichromate (Cr^6+^, CAS: 7778-50-9) were supplied by Aladdin Chemical (Shanghai) Co., Ltd. (Shanghai, China) and the structural formulas are shown in Figure S1 (Supplementary Material).

### 3.2. Preparation of Biochars

BB was carbonized at 500 °C for 60 min with a heating rate of 10 °C/min under the protection of a nitrogen atmosphere to obtain CBB. BB was used by mixing with NaOH and Urea at a ratio of 1:4:1; meanwhile, 1.0 g BB was sufficiently ground with 4.0 g NaOH. The two mixtures were both heated at 700 °C for 60 min. After cooling to room temperature, the activated mixtures were washed with HCl and deionized water until reaching a natural pH value and dried at 180 °C for 12 h. At last, the samples including CBB, BBB, and N-BBB were kept in a desiccator prior to subsequent experiments.

### 3.3. Adsorption Performances

In a batch adsorption experiment, 0.05 g/L BBB or N-BBB was added to a flask containing pollutant solutions (MO or Cr^6+^). The flask was placed in a constant temperature shaker at 150.0 RPM in the dark. After the adsorption process reached equilibrium, the suspension was centrifuged, and the supernatant was diluted with deionized water. The concentration of the solution was determined with an Agilent Cary-300 UV-vis spectrophotometer. The adsorption capacities of samples were calculated by Equation (1):

(1) $$Q_{e}=\frac{(C_{0}-C_{e})\times V}{m}$$

where *Q_e_* (mg/g) represents the adsorption capacity of the sample, *C_e_* represents the equilibrium concentrations of the solution, *C*_0_ (mg/L) represents the initial concentrations of the solution, *m* (g) represents the mass of the samples, and *V* (L) represents the volume of the solutions.

The pollutant solutions were prepared at different concentrations (50, 100, and 200 mg/L). A total of 0.05 g/L BBB or N-BBB was dispersed into flasks containing MO or Cr^6+^ solutions and shaken at 150 RPM in the dark at 303 K. Then, the concentrations of the solutions were determined at preset time intervals. The pseudo-first-order kinetic (PFK, Equation (2)), the pseudo-second-order kinetic (PSK, Equation (3)), and the intra-particle diffusion model (IPD, Equation (4)) were used to analyze the adsorption kinetic data, shown as follows:

(2) $$\ln(Q_{e}-Q_{t})=\ln Q_{e}-k_{1}t$$

(3) $$\frac{t}{Q_{t}}=\frac{1}{k_{2}Q_{t}^{2}}+\frac{t}{Q_{e}}$$

(4) $$Q_{t}=k_{3}t^{0.5}+C$$

where *Q_t_* represents the adsorption capacity of the sample at different time points *t*, *C* represents the thickness of the boundary layer, *k*_1_ represents the PFK adsorption kinetic rate constant, *k*_2_ represents the PSK adsorption kinetic rate constant, and *k*_3_ denotes the IPD adsorption kinetic rate constant.

The pollutant solutions at different initial concentrations (50, 100, 150, 200, and 250 mg/L) were prepared and used to test the adsorption isotherm at 303 K. After adsorption saturation, the absorbances of the solutions were measured using a UV-Vis spectrophotometer. The adsorption isotherm data were investigated using the Langmuir isotherm model (Equation (5)) and Freundlich isotherm model (Equation (6)), as follows:

(5) $$\frac{C_{e}}{Q_{e}}=\frac{C_{e}}{Q_{m}}+\frac{1}{Q_{m}K_{L}}$$

(6) $$\ln Q_{m}=\frac{1}{n}\ln C_{e}+\ln K_{F}$$

where *Q_m_* (mg/g) represents the maximum adsorption capacity of the sample calculated by the adsorption isotherm model, *K_L_* represents the Langmuir adsorption isotherm constant, and *K_F_* represents the Freundlich adsorption isotherm constant.

The effect of temperature (293, 303, and 313 K) on the adsorption capacity of the samples was investigated at an initial concentration of 100 mg/L BBB or 0.05 g/L N-BBB. The thermodynamic parameters were analyzed to describe the effect of temperature on the adsorption process. The calculation equations were as follows:

(7) $$\ln\left(K_{T}\right)=-\frac{\Delta H}{RT}+\frac{\Delta S}{R}$$

(8) $$K_{T}=\frac{Q_{e}}{C_{e}}$$

(9) ΔG=ΔH−TΔS

where Δ*S* represents the thermodynamic parameters’ standard entropy, Δ*G* represents the standard free Gibbs energy, Δ*H* represents the standard enthalpy, and *R* represents the gas constant (8.314 J/K·mol).

The variation in the adsorption capacity of the samples with the pH (2, 4, 6, 8, and 10) was also investigated. The solutions were adjusted to different pH values by HCl and NaOH.

### 3.4. Cycle Tests

In each cycle, 1.0 g/L BBB or N-BBB was placed into a flask containing the organic pollutant solutions at a concentration of 100 mg/L at 303 K. After the adsorption of pollutants (Figure S2, Supplementary Material), the samples, BBB/MO, BBB/Cr^6+^, N-BBB/MO, and N-BBB/Cr^6+^, were collected and washed with deionized water. Then, the recycled samples were carbonized for 60 min at 600 °C under the protection of a nitrogen atmosphere. The re-carbonized samples were re-used as fresh adsorbent in the next cycle.

## 4. Conclusions

In this work, BB was used for the first time to prepare porous biochars via N-doping co-activation. The specific surface area and total pore volume of N-BBB were 2292.7 m^2^/g and 1.0356 cm^3^/g, respectively, which proved the feasibility of N-doping co-activation in pore-forming. The large specific surface area and the high total pore volume played a substantial role in the adsorption process. EDS, FT-IR, and XPS were used to characterize the samples, and the results indicated that nitrogen doping was successfully completed. In an experiment using the synthetic dye MO and heavy metal Cr^6+^ as the pollutant models, N-BBB showed good removal ability. The adsorption capacity of N-BBB remained above 80% after five regenerations, which fully proved the stability of regeneration. Moreover, the excellent adsorption performance of N-BBB may have been influenced by pore filling, π–π interaction, H-bond interaction, and electrostatic attraction. This study not only provided a biochar adsorbent with excellent performance but also verified the feasibility of the one-step nitrogen-doping co-activation method to prepare high-performance biochars. In the future, we will continue to study this N-doped co-activation method and explore its application to other types of biomass to further develop more biochars with better performance.

## Figures and Tables

**Table 1:** The data of N_2_ adsorption-desorption for CBB, BBB, and N-BBB.

Samples	S_BET_ (m^2^/g)	V_micro_ (cm^3^/g)	V_total_ (cm^3^/g)
CBB	49.5	0.0187	0.0222
BBB	2502.3	1.1118	1.1389
N-BBB	2292.7	1.0170	1.0356

S_BET_, V_micro_, and V_total_ represent the BET specific surface area, the volume of micropores, and the total pore volume.

**Table 2:** Fitting parameters of adsorption kinetic models for MO and Cr^6+^ at 303 K.

Adsorbates	Adsorbents	Models	Parameters	*C*_0_ (mg L^−1^)		
				50	100	200
MO	BBB		*Q_e_* (mg/g)	627.9	737.2	836.9
		PFK	*k*_1_ (min^−1^)	0.0028	0.0074	0.0058
			*Q_e.cat_* (mg/g)	611.8	720.4	800.3
			*R* ^2^	0.9880	0.9944	0.9780
		PSK	*k*_2_ (g mg^−1^ min^−1^)	0.0003	0.0003	0.0005
			*Q_e.cat_* (mg/g)	664.5	769.0	844.2
			*R* ^2^	0.9848	0.9896	0.9997
		IPD	*k*_3_ (mg g^−1^ min^−0.5^)	27.12	23.62	19.90
			*C*	350.1	497.9	628.2
			*R* ^2^	0.6464	0.5822	0.7517
	N-BBB		*Q_e_* (mg/g)	644.4	755.1	858.3
		PFK	*k*_1_ (min^−1^)	0.0044	0.0054	0.0051
			*Q_e.cat_* (mg/g)	631.3	732.9	817.1
			*R* ^2^	0.9952	0.9885	0.9721
		PSK	*k*_2_ (g mg^−1^ min^−1^)	0.0004	0.0004	0.0005
			*Q_e.cat_* (mg/g)	672.1	777.9	862.8
			*R* ^2^	0.9873	0.9980	0.9989
		IPD	*k*_3_ (mg g^−1^ min^−0.5^)	19.4	22.0	20.9
			*C*	448.7	530.3	637.3
			*R* ^2^	0.5471	0.6549	0.7830
Cr^6+^	BBB		*Q_e_* (mg/g)	93.8	119.6	141.1
		PFK	*k*_1_ (min^−1^)	0.0014	0.0017	0.0020
			*Q_e.cat_* (mg/g)	85.3	109.2	130.4
			*R* ^2^	0.9741	0.9614	0.9550
		PSK	*k*_2_ (g mg^−1^ min^−1^)	0.0007	0.0008	0.0008
			*Q_e.cat_* (mg/g)	98.4	122.3	143.6
			*R* ^2^	0.9923	0.9919	0.9913
		IPD	*k*_3_ (mg g^−1^ min^−0.5^)	6.2	6.9	7.3
			*C*	22.7	41.4	59.1
			*R* ^2^	0.9138	0.9280	0.9279
	N-BBB		*Q_e_* (mg/g)	99.5	135.0	169.1
		PFK	*k*_1_ (min^−1^)	0.0015	0.0017	0.0017
			*Q_e.cat_* (mg/g)	89.7	122.4	153.0
			*R* ^2^	0.9500	0.9506	0.9318
		PSK	*k*_2_ (g mg^−1^ min^−1^)	0.0007	0.0007	0.0006
			*Q_e.cat_* (mg/g)	102.2	135.9	170.6
			*R* ^2^	0.9843	0.9869	0.9765
		IPD	*k*_3_ (mg g^−1^ min^−0.5^)	6.2	7.3	9.6
			*C*	27.7	50.5	58.4
			*R* ^2^	0.9537	0.9432	0.9746

**Table 3:** Fitting parameters of adsorption isotherm models for MO and Cr^6+^ at 303 K.

Adsorbates	Adsorbents	Types	Parameters	
MO	BBB	Langmuir	*Q_m_* (mg/g)	860.3
			*K_L_* (L/mg)	0.1345
			*R* ^2^	0.9327
		Freundlich	*K_F_* (mg g^−1^(L mg^−1^)^1/n^)	431.5
			*n_F_*	7.75
			*R* ^2^	0.9967
	N-BBB	Langmuir	*Q_m_* (mg/g)	887.4
			*K_L_* (L/mg)	0.1364
			*R* ^2^	0.9041
		Freundlich	*K_F_* (mg g^−1^(L mg^−1^)^1/n^)	437.1
			*n_F_*	7.51
			*R* ^2^	0.9883
Cr^6+^	BBB	Langmuir	*Q_m_* (mg/g)	169.8
			*K_L_* (L/mg)	0.0264
			*R* ^2^	0.9949
		Freundlich	*K_F_* (mg g^−1^(L mg^−1^)^1/n^)	34.9
			*n_F_*	3.76
			*R* ^2^	0.9976
	N-BBB	Langmuir	*Q_m_* (mg/g)	226.2
			*K_L_* (L/mg)	0.0163
			*R* ^2^	0.9822
		Freundlich	*K_F_* (mg g^−1^(L mg^−1^)^1/n^)	25.7
			*n_F_*	2.77
			*R* ^2^	0.9959

**Table 4:** Fitting adsorption thermodynamic parameters for MO and Cr^6+^.

Adsorbents	Adsorbates	T (K)	∆*G* (kJ/mol)	∆*H* (kJ/mol)	∆*S* (J mol^−1^ K^−1^)
BBB	MO	293	−5.86	4.29	34.61
		303	−6.19		
		313	−6.55		
	Cr^6+^	293	−0.27	6.47	23.01
		303	−0.61		
		313	−0.73		
N-BBB	MO	293	−5.93	3.82	33.27
		303	−6.29		
		313	−6.60		
	Cr^6+^	293	−0.43	11.23	39.78
		303	−0.93		
		313	−123		

**Table 5:** Comparison of BBB and N-BBB to MO and Cr^6+^ with other biochars.

Adsorbents	*Q_e_* for MO (mg/g)	*Q_e_* for Cr^6+^ (mg/g)	References
BBB	836.9	141.1	This work
N-BBB	858.3	169.1	This work
Pomelo peel biochar	147.9	-	[46]
Magnetic bamboo biochar	305.4	-	[47]
Date seeds biochar	334.0	-	[48]
Lotus root biochar	449.0	-	[49]
Date palm petioles biochar	461.0	-	[50]
Landfill leachate sludge biochar	-	17.5	[51]
Zn/iron-based sludge/biochar	-	27.0	[52]
*Potamogeton crispus* biochar	-	34.4	[53]
*Egeria najas* biochar	-	138.8	[54]
Soybean protein biochar	-	489.7	[55]

## Supplementary Materials

The following supporting information can be downloaded at: https://www.mdpi.com/article/10.3390/ijms232314618/s1.

## References

[1] Zhou L., Wu Y., Zhang S., Li Y., Gao Y., Zhang W., Tian L., Li T., Du Q., Sun S. (2022). Recent development in microbial electrochemical technologies: Biofilm formation, regulation, and application in water pollution prevention and control. J. Water Process Eng..

[2] Yan C., Qu Z., Wang J., Cao L., Han Q. (2022). Microalgal bioremediation of heavy metal pollution in water: Recent advances, challenges, and prospects. Chemosphere.

[3] Zamora-Ledezma C., Negrete-Bolagay D., Figueroa F., Zamora-Ledezma E., Ni M., Alexis F., Guerrero V.H. (2021). Heavy metal water pollution: A fresh look about hazards, novel and conventional remediation methods. Environ. Technol. Innov..

[4] Xu H., Fan Y., Xia X., Liu Z., Yang S. (2023). Effect of Ginkgo biloba leaves on the removal efficiency of Cr(VI) in soil and its underlying mechanism. Environ. Res..

[5] Njoya O., Zhao S., Shen J., Kong X., Gong Y., Wang B., Kang J., Chen Z. (2022). Acetate improves catalytic performance for rapid removal of Cr(VI) by sodium borohydride in aqueous environments. Sep. Purif. Technol..

[6] Li X.Q., Zhang Q.H., Ma K., Li H.M., Guo Z. (2015). Identification and determination of 34 water-soluble synthetic dyes infoodstuff by high performance liquid chromatography–diode arraydetection–ion trap time–of–flight tandem mass spectrometry. Food Chem..

[7] Moghadas M.R.S., Motamedi E., Nasiri J., Naghavi M.R., Sabokdast M. (2020). Proficient dye removal from water using biogenic silver nanoparticles prepared through solid-state synthetic route. Heliyon.

[8] Mandal B., Ray S.K. (2013). Synthesis of interpenetrating network hydrogel from poly(acrylic acid-co-hydroxyethyl methacrylate) and sodium alginate: Modeling and kinetics study for removal of synthetic dyes from water. Carbohyd. Polym..

[9] Baig U., Kashif Uddin M., Gondal M.A. (2020). Removal of hazardous azo dye from water using synthetic nano adsorbent: Facile synthesis, characterization, adsorption, regeneration and design of experiments. Colloids Surf. A.

[10] Nidheesh P.V., Zhou M., Oturan M.A. (2018). An overview on the removal of synthetic dyes from water by electrochemical advanced oxidation processes. Chemosphere.

[11] Ambika, Kumar V., Jamwal A., Kumar V., Singh D. (2022). Green bioprocess for degradation of synthetic dyes mixture using consortium of laccase-producing bacteria from Himalayan niches. J. Environ. Manag..

[12] Zaafouri Z., Batot G., Nieto-Draghi C., Coasne B., Bauer D. (2022). Impact of adsorption kinetics on pollutant dispersion in water flowing in nanopores: A Lattice Boltzmann approach to stationary and transient conditions. Adv. Water Resour..

[13] Tee G.T., Gok X.Y., Yong W.F. (2022). Adsorption of pollutants in wastewater via biosorbents, nanoparticles and magnetic biosorbents: A review. Environ. Res..

[14] Zhou W., Zhang W., Cai Y. (2022). Enzyme-enhanced adsorption of laccase immobilized graphene oxide for micro-pollutant removal. Sep. Purif. Technol..

[15] Ma Y., Li Y., Zhao X., Zhang L., Wang B., Nie A., Mu C., Xiang J., Zhai K., Xue T. (2022). Lightweight and multifunctional super-hydrophobic aramid nanofiber/multiwalled carbon nanotubes/Fe_3_O_4_ aerogel for microwave absorption, thermal insulation and pollutants adsorption. J. Alloys Compd..

[16] Sellaoui L., Gomez-Aviles A., Dhaouadi F., Bedia J., Bonilla-Petriciolet A., Rtimi S., Belver C. (2023). Adsorption of emerging pollutants on lignin-based activated carbon: Analysis of adsorption mechanism via characterization, kinetics and equilibrium studies. Chem. Eng. J..

[17] Qiu B., Shao Q., Shi J., Yang C., Chu H. (2022). Application of biochar for the adsorption of organic pollutants from wastewater: Modification strategies, mechanisms and challenges. Sep. Purif. Technol..

[18] Zhang B., Wang M., Qu J., Zhang Y., Liu H. (2021). Characterization and mechanism analysis of tylosin biodegradation and simultaneous ammonia nitrogen removal with strain Klebsiella pneumoniae TN-1. Bioresour. Technol..

[19] Wu J., Wang T., Liu Y., Tang W., Geng S., Chen J. (2022). Norfloxacin adsorption and subsequent degradation on ball-milling tailored N-doped biochar. Chemosphere.

[20] Qu J., Zhang X., Liu S., Li X., Wang S., Feng Z., Wu Z., Wang L., Jiang Z., Zhang Y. (2022). One-step preparation of Fe/N co-doped porous biochar for chromium(VI) and bisphenol a decontamination in water: Insights to co-activation and adsorption mechanisms. Bioresour. Technol..

[21] Li J., Lin Q., Luo H., Fu H., Wu L., Chen Y., Ma Y. (2022). The effect of nanoscale zero-valent iron-loaded N-doped biochar on the generation of free radicals and nonradicals by peroxydisulfate activation. J. Water Process Eng..

[22] Tan X., Zhu S., Wang R., Chen Y., Showf P., Zhang F., Ho S. (2021). Role of biochar surface characteristics in the adsorption of aromatic compounds: Pore structure and functional groups. Chin. Chem. Lett..

[23] Zhang J., Huang D., Shao J., Zhang X., Yang H., Zhang S., Chen H. (2022). Activation-free synthesis of nitrogen-doped biochar for enhanced adsorption of CO_2_. J. Clean. Prod..

[24] Liew R., Azwar E., Yek P., Lim X., Cheng C., Ng J., Jusoh A., Lam W., Ibrahim M., Ma N. (2018). Microwave pyrolysis with KOH/NaOH mixture activation: A new approach to produce micro-mesoporous activated carbon for textile dye adsorption. Bioresour. Technol..

[25] Chen S., Zhang B., Xia Y., Chen H., Chen G., Tang S. (2021). Influence of mixed alkali on the preparation of edible fungus substrate porous carbon material and its application for the removal of dye. Colloids Surf. A.

[26] Wei M., Marrakchi F., Yuan C., Cheng X., Jiang D., Zafar F.F., Fu Y., Wang S. (2022). Adsorption modeling, thermodynamics, and DFT simulation of tetracycline onto mesoporous and high-surface-area NaOH-activated macroalgae carbon. J. Hazard. Mater..

[27] Alfatah T., Mistar E.M., Supardan M.D. (2021). Porous structure and adsorptive properties of activated carbon derived from Bambusa vulgaris striata by two-stage KOH/NaOH mixture activation for Hg^2+^ removal. J. Water Process Eng..

[28] Kamran U., Park S. (2020). Tuning ratios of KOH and NaOH on acetic acid-mediated chitosan-based porous carbons for improving their textural features and CO_2_ uptakes. J. CO_2_ Util..

[29] Yang X., Wang Q., Lai J., Cai Z., Lv J., Chen X., Chen Y., Zheng X., Huang B., Lin G. (2020). Nitrogen-doped activated carbons via melamine-assisted NaOH/KOH/urea aqueous system for high performance supercapacitors. Mater. Chem. Phys..

[30] Hou Z., Tao Y., Bai T., Liang Y., Huang S., Cai J. (2021). Efficient Rhodamine B removal by N-doped hierarchical carbons obtained from KOH activation and urea-oxidation of glucose hydrochar. J. Environ. Chem. Eng..

[31] Wang K., Xu M., Gu Y., Gu Z., Fan Q.H. (2016). Symmetric supercapacitors using urea-modified lignin derived Ndoped porous carbon as electrode materials in liquid and solid electrolytes. J. Power Sources.

[32] Jin Y., Zhang B., Chen G., Chen H., Tang S. (2022). Combining biological and chemical methods to disassemble of cellulose from corn straw for the preparation of porous carbons with enhanced adsorption performance. Int. J. Biol. Macromol..

[33] Chen S., Xia Y., Zhang B., Chen H., Chen G., Tang S. (2021). Disassembly of lignocellulose into cellulose, hemicellulose, and lignin for preparation of porous carbon materials with enhanced performances. J. Hazard. Mater..

[34] Zhang B., Jin Y., Qi J., Chen H., Chen G., Tang S. (2021). Porous carbon materials based on *Physalis alkekengi* L. husk and its application for removal of malachite green. Environ. Technol. Innov..

[35] Xia Y., Jin Y., Qi J., Chen H., Chen G., Tang S. (2021). Preparation of biomass carbon material based on Fomes fomentarius via alkali activation and its application for the removal of brilliant green in wastewater. Environ. Technol. Innov..

[36] Fan C., Yan J., Huang Y., Han X., Jiang X. (2015). XRD and TG-FTIR study of the effect of mineral matrix on the pyrolysis and combustion of organic matter in shale char. Fuel.

[37] Yan J., Jiang X., Han X., Liu J. (2013). A TG–FTIR investigation to the catalytic effect of mineral matrix in oil shaleon the pyrolysis and combustion of kerogen. Fuel.

[38] Zhang B., Jin Y., Huang X., Tang S., Chen H., Su Y., Yu X., Chen S., Chen G. (2022). Biological self-assembled hyphae/starch porous carbon composites for removal of organic pollutants from water. Chem. Eng. J..

[39] Xia Y., Zhang B., Guo Z., Tang S., Su Y., Yu X., Chen S., Chen G. (2022). Fungal mycelium modified hierarchical porous carbon with enhanced performance and its application for removal of organic pollutants. J. Environ. Chem. Eng..

[40] Chen X., Yu G., Chen Y., Tang S., Su Y. (2022). Cow dung-based biochar materials prepared via mixed base and its application in the removal of organic pollutants. Int. J. Mol. Sci..

[41] Chauhdary Y., Hanif M.A., Rashid U., Bhatti I.A., Anwar H., Jamil Y., Alharthi F.A., Kazerooni E.A. (2022). Effective removal of reactive and direct dyes from colored wastewater using low-cost novel bentonite nanocomposites. Water.

[42] Lin L., Li L., Xiao L., Zhang C., Li X., Pervez M.N., Zhang Y., Nuruzzaman M., Mondal M.I.H., Cai Y. (2022). Adsorption behaviour of reactive blue 194 on raw Ramie Yarn in palm oil and water media. Materials.

[43] El-Sayed N.S., Salama A., Guarino V. (2022). Coupling of 3-Aminopropyl sulfonic acid to cellulose nanofifibers for effificient removal of cationic dyes. Materials.

[44] Shirendev N., Bat-Amgalan M., Kano N., Kim H.-J., Gunchin B., Ganbat B., Yunden G. (2022). A natural zeolite developed with 3-Aminopropyltriethoxylane and adsorption of Cu(II) from aqueous media. Appl. Sci..

[45] Jiao Y., Xu L., Sun H., Deng Y., Zhang T., Liu G. (2017). Synthesis of benzxazine-based nitrogen-doped mesoporous carbon spheres for methyl orange dye adsorption. J. Porous Mater..

[46] Zhang B., Wu Y., Cha L. (2018). Removal of methyl orange dye using activated biochar derived from pomelo peel wastes: Performance, isotherm, and kinetic studies. J. Disper. Sci. Technol..

[47] Zhang H., Li R., Zhang Z. (2022). A versatile EDTA and chitosan bi-functionalized magnetic bamboo biochar for simultaneous removal of methyl orange and heavy metals from complex wastewater. Environ. Pollut..

[48] Ouedrhiri A., Himi M.A., Youbi B., Lghazi Y., Bahar J., Haimer C.E., Aynaou A., Bimaghra I. (2022). Biochar material derived from natural waste with superior dye adsorption performance. Mater. Today. Proc..

[49] Hou Y., Liang Y., Hu H., Tao Y., Zhou J., Cai J. (2021). Facile preparation of multi-porous biochar from lotus biomass for methyl orange removal: Kinetics, isotherms, and regeneration studies. Bioresour. Technol..

[50] Aichour A., Zaghouane-Boudiaf H., Khodja H.D. (2022). Highly removal of anionic dye from aqueous medium using a promising biochar derived from date palm petioles: Characterization, adsorption properties and reuse studies. Arab. J. Chem..

[51] Li Y., Chen X., Liu L., Liu P., Zhou Z., Huhetaoli, Wu Y., Lei T. (2022). Characteristics and adsorption of Cr(VI) of biochar pyrolyzed from landfill leachate sludge. J. Anal. Appl. Pyrolysis.

[52] Zhen Z., Duan X., Tie J. (2022). One-pot synthesis of a magnetic Zn/iron-based sludge/biochar composite for aqueous Cr(VI) adsorption. Environ. Technol. Innov..

[53] Xu D., Sun T., Jia H., Sun Y., Zhu X. (2022). The performance and mechanism of Cr(VI) adsorption by biochar derived from Potamogeton crispus at different pyrolysis temperatures. J. Anal. Appl. Pyrolysis.

[54] Yi Y., Wang X., Zhang Y., Ma J., Ning P. (2022). Adsorption properties and mechanism of Cr(VI) by Fe_2_(SO_4_)_3_ modified biochar derived from *Egeria najas*. Colloids Surf. A.

[55] Kuang Q., Liu K., Wang Q., Chang Q. (2023). Three-dimensional hierarchical pore biochar prepared from soybean protein and its excellent Cr(VI) adsorption. Sep. Purif. Technol..

