# Supplementary material for: One-Step Synthesis of Nitrogen-Doped Porous Biochar Based on N-Doping Co-Activation Method and Its Application in Water Pollutants Control
Source: Int J Mol Sci. 2022 Nov 23;23(23):14618. doi: 10.3390/ijms232314618 (PMC9739037; doi:10.3390/ijms232314618)
Supplement: Supplementary file 1 [file ijms-23-14618-s001.zip › ijms-2006355-supplementary.pdf]

One-Step Synthesis of Nitrogen-Doped Porous Biochar Based on N-Doping Co-Activation Method and Its Application in Water Pollutants Control

Yingjie Su [1,2], Yuqing Shi [1,2], Meiyi Jiang [1,2], Siji Chen [1,2,*]

[1] College of Life Sciences, Jilin Agricultural University, Changchun 130118, China

[2] Key Laboratory of Straw Comprehensive Utilization and Black Soil Conservation, Ministry of Education, Jilin Agricultural University, Changchun 130118, China

* Corresponding author E-mail: 18638342679@163.com.

S1 Characterization methods

Scanning electron microscopy (SEM, Hitachi S4800, Japan) was used to examine the morphology of materials. Thermogravimetric analysis of the samples was carried out under the protection of nitrogen flow (TGA, Netzsch STA409PC, Germany). FT-IR spectrometer was used to characterize the surface functional groups of materials between 400 and 4000 cm^{-1} at a resolution of 1 cm^{-1} (FT-IR, Thermo Fisher Nicolet iS50, USA). X-ray diffraction patterns of the powders were observed by an X-ray diffractometer with a filtered Cu-Ka X-ray source (XRD, Bruker D8 Advance, Germany). Raman spectra of the samples were obtained using a model Renishaw 2000 Raman spectrometer at 514 nm to investigate the presence of defects in the biochar materials. X-ray photoelectron spectroscopy was used to test the electronic binding energy of the samples (XPS, Thermo Escalab $250Xi^{+}$, USA). The zeta potential instrument was used to characterize the surface charge of samples (Zeta potential, Zetasizer Nano ZS90, UK). N_2 adsorption-desorption isotherms were used to obtain the porosity of the samples at 77 K (N_2 adsorption-desorption isotherms, Quantachrome Autosorb iQ2, USA). The Brunauer-Emmett-Teller (BET) theory was used to calculate the surface area. The non-local density functional theory (NLDFT), HK method (HK), and the Barrett-Joyner-Halenda (BJH) model were used to analyse

the pore size distribution of samples. The zeta potential instrument was used to characterize the surface charge of samples (Zeta potential, Zetasizer Nano ZS90, UK).

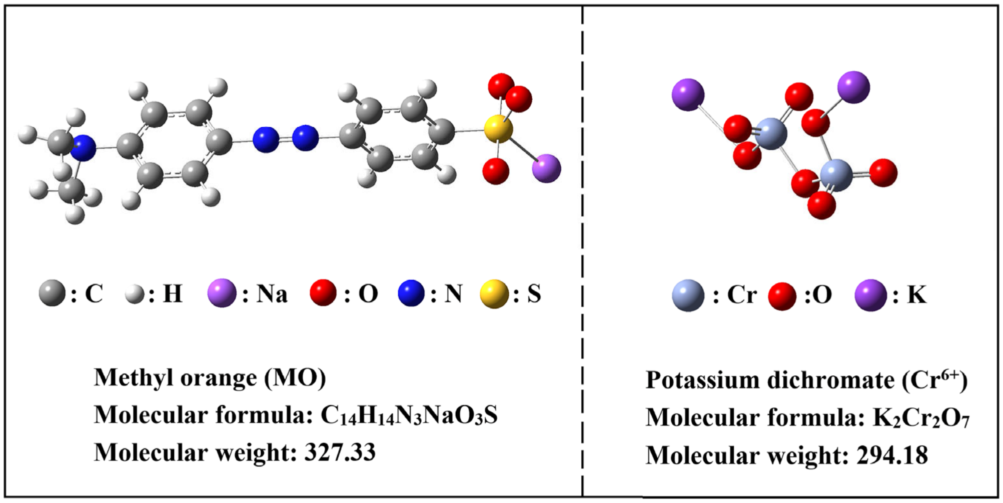

Figure S1. The structural formulas of MO and Cr^{6+}.

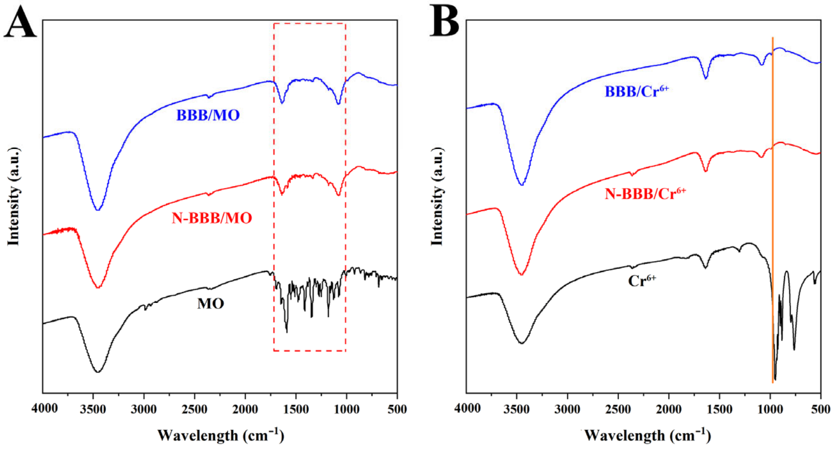

Figure S2. FT-IR spectra of biochars after the adsorption of pollutants.
